# GSK3β Inhibition Promotes Synaptogenesis in *Drosophila* and Mammalian Neurons

**DOI:** 10.1371/journal.pone.0118475

**Published:** 2015-03-12

**Authors:** Germán Cuesto, Sheila Jordán-Álvarez, Lilian Enriquez-Barreto, Alberto Ferrús, Miguel Morales, Ángel Acebes

**Affiliations:** 1 Structural Synaptic Plasticity Laboratory, Department of Neurodegenerative Diseases, Centro de Investigación Biomédica de La Rioja, Logroño, La Rioja, Spain; 2 Department of Cellular, Molecular and Developmental Neurobiology, Cajal Institute, Consejo Superior de Investigaciones Científicas, Madrid, Spain; University of Florida, UNITED STATES

## Abstract

The PI3K-dependent activation of AKT results in the inhibition of GSK3β in most signaling pathways. These kinases regulate multiple neuronal processes including the control of synapse number as shown for *Drosophila* and rodents. Alzheimer disease’s patients exhibit high levels of circulating GSK3β and, consequently, pharmacological strategies based on GSK3β antagonists have been designed. The approach, however, has yielded inconclusive results so far. Here, we carried out a comparative study in *Drosophila* and rats addressing the role of GSK3β in synaptogenesis. In flies, the genetic inhibition of the *shaggy*-encoded GSK3β increases the number of synapses, while its upregulation leads to synapse loss. Likewise, in three weeks cultured rat hippocampal neurons, the pharmacological inhibition of GSK3β increases synapse density and Synapsin expression. However, experiments on younger cultures (12 days) yielded an opposite effect, a reduction of synapse density. This unexpected finding seems to unveil an age- and dosage-dependent differential response of mammalian neurons to the stimulation/inhibition of GSK3β, a feature that must be considered in the context of human adult neurogenesis and pharmacological treatments for Alzheimer’s disease based on GSK3β antagonists.

## Introduction

Glycogen synthase kinase 3 (GSK3) [1,2] is a serine/threonine kinase that regulates neuronal polarization, neuritogenesis [3], migration, axon growth and guidance [4–8] among other cellular processes. To date, two GSK3 genes (GSK3α and GSK3β) and a neuronal variant of GSK3β (GSK3β2), [9] have been identified in mammals [10–11]. In rodent adult hippocampus, GSK3β is more abundant than GSK3α [12] and it increases the most in aged brains [13]. In *Drosophila* larvae, GSK3β is represented by two splice variants (Sgg10 and Sgg39) encoded in the single gene *shaggy* (*sgg*) [14–15]. In this study, all genetic and pharmacological experiments are referred to GSK3β activity.

The functional requirements of GSK3 are diverse and, consequently, the repertoire of elicited pathologies is extensive. Focusing on the nervous system, in mammalian brains, GSK3 is required for proliferation, differentiation and neurotransmission in adult neurons [16–20]. GSK3 is detected at both pre- and postsynaptic compartments controlling endocytosis of synaptic vesicles and hence, neurotransmission [21]. In neuronal networks, GSK3 participates in long-term potentiation and depression (LTP/LTD) [19,22]. Changes in GSK3 levels underlie pathologies as diverse as muscular dystrophy, cancer and bipolar disorders, as well as autism, schizophrenia, Fragile X syndrome or Alzheimer’s diseases (AD) [23–26]. AD patients show high levels of circulating GSK3 [9,27], which has led to GSK3 inhibitor-based strategies to treat the disease. In spite of this sound rationale, however, the results have not been positive. Here, we set out a comparative study of the synaptic effects of GSK3 inhibition in *Drosophila* and *Rattus*, aimed to identify possible reasons for the puzzling failure of current AD pharmacological approaches.

We have previously identified a phosphoinositide 3-kinase (PI3K) synaptogenic pathway in *Drosophila* which is conserved in vertebrates. Activation/inhibition of PI3K or downstream elements of this pathway coherently regulate the number of synapses [28–29]. While the overactivation of PI3K-AKT signaling results in functional supernumerary synapses both in *Drosophila* and mammals [28–30], the overexpression of GSK3 causes a reduction of synapse number [28,31–32]. The pathway includes also the Jun kinase/AP-1 and Wnt signaling which are modulated by GSK3 [33]. Likewise, in the granular cells of the rodent cerebellum, Wnt regulates Synapsin clustering through a Dvl/GSK3 dependent mechanism [34]. Indirect evidences indicate that GSK3 inhibitors increase Synapsin clustering [7] whereas transient GSK3 overexpression decreases Synapsin expression [22].

The molecular mechanisms that sustain these cellular processes involve changes in the phosphorylation status of specific residues in these kinases. Thus, after PI3K activation, AKT is phosphorylated in Serine 473 by mTORC2 and in Threonine 308 by PDK1 [35]; which results in the inhibition of GSK3 by phosphorylation of its Serine 9 [1]. However, GSK3 is an unusual kinase in the sense that it has a high basal activity at resting conditions. Its activity is down-regulated by upstream elements in its pathway [36], other kinases, or the phosphorylation status of their multiple substrates [1,37]. These features could be expected to result in heterogeneous responses following the experimental manipulation of GSK3 activity.

Here, we show that the downregulation of GSK3 promotes synapse formation in *Drosophila* neurons. In rodent hippocampal neurons, however, GSK3 inhibition yields differential results according to cell culture age and dosage. Young culture neurons show a decrease of synapses while aged cultures respond with an increase of synapse number. Also, whereas spine density increases under low concentrations of GSK3 inhibitor, high concentrations reduce the number of spines.

## Materials and Methods

### Animals

#### Flies:

Line *D42-Gal4* was kindly provided by H. L Atwood (University of Toronto) [38]. Line *elav-Gal4* [39], *UAS-AKT* [40] and *UAS-GSK3* ^*DN*^ [41] were obtained from Bloomington Stock Center. The *UAS-PI3K92E* (referred here as UAS-PI3K) and *UAS-PI3K92E* ^*D954A*^ (UAS-PI3K^DN^) stocks originate from S. Leevers (Cancer Research Center, London). The *UAS-GSK3* ^*RNAi*^ construct was obtained from the Vienna Stock Center (http://stockcenter.vdrc.at/control/main) (reference 101538KK) and the *UAS-GSK3* strain was provided by Dr. Manuel Calleja (Centro de Biología Molecular, Madrid) [15].

#### Rodent neurons:

Primary hippocampal cultures were obtained from P0 rat pups (Sprague-Dawley, strain, Harlan Laboratories Models SL, France). Animals were anesthetized by hypothermia in paper-lined towel over crushed-ice surface during 2–4 minutes and euthanized by decapitation. Animals were handled and maintained in accordance with the Council Directive guidelines 2010/63EU of the European Parliament, and approved by the Ethical Committee of the CIBIR.

### Cell cultures

#### SH-SY5Y cell cultures:

SH-SY5Y human neuroblastoma cells were purchased from ATCC (ref: CRL-2266). Cells were seeded at 5x10^4^ cells/cm^2^ and used 5 days later, usually when cultures reached a 70–80% confluence. Culture media contained DMEM F-12 Ham (Sigma-Aldrich, USA) supplemented with 0.5 mM glutamine (Sigma-Aldrich, USA), penicillin (50 mg/ml)/streptomycin (50 U/ml) from Sigma-Aldrich (USA), and 10% FBS (Sigma-Aldrich, USA). Cells were serum starved for 16 hours prior to treatment, in order to reduce Akt basal activity.

#### Hippocampal neuron cultures:

Primary cultures of hippocampus neurons were prepared as previously described [42–43]. Glass coverslips (12 mm in diameter) were coated with poly-L-lysine (100 μg/ml) and laminin (4 μg/ml). Hippocampus neurons were seeded and grown in Neurobasal (Invitrogen, USA) culture medium supplemented with glutamine 0.5 mM, 50 mg/ml penicillin, 50 units/ml streptomycin, 4% FBS and 4% B27 (Invitrogen, CA, USA), as described before [29]. After 4, 7, 14 and 21 days in culture, 100 μl (of a total of 500 μl) of culture medium was replaced by 120 μl of fresh medium. On day 4th, 4 μM cytosine-D-arabinofuranoside was added to prevent overgrowth of glial cells. Two seeding densities were employed: a medium-low density of 10x10^4^ neurons/cm^2^ for immunocytochemistry, and a higher density of 50x10^4^ neurons/cm^2^ for biochemical experiments. In both cases, cultured cells were seeded on plastic 24-wells plates.

#### Immunohistochemistry, image acquisition and quantification of *Drosophila* synapse number

We systematically used the nc82 Mab to identify the active zone component, CAST/Bruchpilot, of synapses. The matching between presynaptic nc82 and postsynaptic GluRII immunosignals have been previously documented [44]. Late third instar larvae were dissected, fixed and processed as previously described [44]. Specimens were incubated overnight at 4°C in blocking solution with the following primary antibodies: monoclonal antibody nc82 (DSHB, USA, 1:10) to count individual synapses, and anti-HRP (Jackson ImmunoResearch Laboratories, USA, 1:200), to visualize the neuronal membrane. The following secondary antibodies were applied for 3 hours at room temperature: Alexa 488 (goat anti-mouse, Molecular Probes, USA, 1:500) and Alexa 568 (goat anti-rabbit, Molecular Probes, USA, 1:500). Larvae were mounted in Vectashield (Vector Labs, USA). Confocal Images from muscle fibers 6–7 (segment A3) were acquired with a Leica Confocal Microscope TCS SP5 II (Mannheim, Germany). Serial optical sections at 1024x512 pixels (100x50 mm) or 1024x1024 pixels (100x100 mm) were obtained at 0.5 μm with the 63x objective. Image J software (version 1.44, http://rsb.info.nih.gov/ij/, NIH, USA) was used to determine the number of synapses per neuromuscular junction (NMJ). Individual synapses (nc82 positive puncta) were quantified by using the Point Picker plug-in as published previously [40]. A minimum of 10–12 larvae from each genotype were analyzed.

#### Cell culture immunocytochemistry

Synaptic density on hippocampal cultures was analyzed as previously described [29]. In short, cultures were rinsed in phosphate buffer saline (PBS) and fixed for 30 min in 4% paraformaldehyde-PBS. Coverslips were incubated overnight in blocking solution with the following antibodies: anti-Bassoon monoclonal mouse antibody (ref. VAM-PS003, Stress Gen, USA) and rabbit polyclonal sera against Synapsin (ref. 2312, Cell Signaling, USA). Samples were incubated with a fluorescence-conjugated secondary antibody in PBS for 30 min. After that, coverslips were washed three times in PBS and mounted using Mowiol (all secondary antibodies from Molecular Probes-Invitrogen, USA). Stack images (pixel size 90 nm with 0.5 μm Z step) were obtained with a Leica SP5 Confocal microscope. Image J (version 1.47) “deconvolution Acoloma macro” plugin was used to process the images before analysis. Synaptic density and dendritic length quantification was done manually with Image J software. To reduce variability among different cultures and treatments, synaptic puncta were counted in proximal dendrites only, starting from a clearly identified neuronal cell soma. Synaptic density represents the number of synapses per 100 μm of dendritic length. Percentage of synaptic change is the average of different cultures under the same experimental conditions. As a control, we used sister untreated cultures growing in the same multi-well plate.

#### Transfection and image analysis of spines in rat hippocampal cultures

Neuronal GFP-Actin expression was obtained by electroporation before plating, employing a BioRad Cell electroporator system. Approximately 4x10^6^ cells and 10 μg of plasmid were mixed in BioRad electroporation buffer (BioRad). An exponential discharge protocol (220 V, 950 μF, resistance fixed to infinitum) was employed. Spines were visualized by transfection with a plasmid encoding the GFP protein fused to chick β-actin under the control of the platelet-derived growth factor promoter region (kindly provided by Y. Goda, MRC Cell Biology Unit, University College London, London, UK) [45]. Transfected cultures were fixed with 4% paraformaldehyde-PBS, washed and mounted in Mowiol. Images were obtained in a Leica SP5 Confocal, acquired in stacks (pixel size 60 nm with 0.1 μm Z step) and processed as above. Spine density represents the number of spines per 100 μm of dendritic length.

#### Western blots

For *Drosophila* samples, nitrocellulose membranes were incubated with the following primary antibodies: mouse anti-GSK3 (detecting both *Drosophila* GSK3 splice variants) (ref. 05-412, Millipore, USA; 1:1000), rabbit anti-AKT (ref. 9272, Cell Signaling, USA, 1:1000) and rabbit anti-phosporylated-AKT (p-S505AKT) (ref. 4054, Cell Signaling, USA, 1:1000), with 3% non-fat dry milk (Bio-Rad) in 0.05% PBST (PBS + Tween 20, Sigma-Aldrich) overnight at 4°C. They were washed with double distilled water twice and incubated with secondary peroxidase-conjugated antibodies goat anti-mouse or goat anti-rabbit IgG (ref. A9044 and A0545, Sigma-Aldrich, USA, 1:5000) for 1.5 hours at room temperature. Membranes were washed with PBST (2x10 minutes) and revealed with SuperSignal (Thermo Scientific). Western blots of *Drosophila* and rat materials were quantified as previously described [24,44]. Bands were scanned using a GS800 densitometer and Quantity One software (Bio-Rad). All measurements were normalized to tubulin. Graphs represent the average of three independent Western blot experiments.

The same Western blot protocol was employed for SH-SY5Y and neuronal cultures. Briefly, cell wells were washed with PBS (4°C) and scrapped in 60 μl of lysis buffer (Cell Lysis Buffer, ref. 9803, Cell Signaling). Once collected, cell lysates were heated, sonicated and clarified by centrifugation at 17000 g. Protein concentration was measured using Bradford protein assay and 60 μg of total protein were loaded into each SDS-PAGE lane. After blotting, membranes were incubated overnight with the following antibodies: anti-Actin (ref. A2547, Sigma), anti-GSK3 α/β (ref. 44-610, Invitrogen), anti-phospho-S9GSK3β (ref. 2435-1, Epitomics), anti-Synapsin I (ref. 160 001, Synaptic Systems), anti-AKT (pan) (C67E7) (ref. 4691, Cell Signaling) and the following polyclonal antibodies: anti-phospho-S473AKT1 (ref. NB600-590, Novus Biologicals), anti-β-Catenin (ref. 9562, Cell Signaling) and anti-phospho-β-Catenin (Ser33/37/Thr41) (ref. 9561, Cell Signaling). Signal was detected using the corresponding IR-fluorescence-labelled antibodies (Li-Cor Bioscience, USA) and visualized and quantified with an Odyssey Infrared Imaging System (Li-Cor Bioscience). Unless otherwise indicated, all biochemical experiments were performed adding the peptide at day 10 or 19 in vitro, cell lysis was done 48 hours later. The p-S473AKT and p-S9GSK3β values were related to the total AKT and GSK3β, respectively. Synapsin and phospho-β-Catenin levels were related to Actin. Average changes in phosphorylation and percentage of protein represent the normalized value with respect to the control value, always included in the same Western-blot membrane. Values over 100 indicate increase in protein levels and values below 100 indicate reduction.

#### PI3K activating peptides and GSK3 inhibitors

Activation of PI3K was achieved by a transduction peptide (PTD4-PI3KAc) with the following sequence: YARAAARQARAGSDGGpYMDMS [29,46–48]. This is a cell membrane permeable phosphopeptide composed of a transduction domain of the tat family [48] fused to a SH2 interacting domain that allows the activation of the class I PI3K independently from tyrosine kinase dimerization [29,48–49]. Peptides were purchased either from GenScript (NJ, USA) or BioPeptide (FR). GSK3 inhibitors: SB-415286 and AR-A014418 [50–52] were from Tocris, UK (ref. 1617) and from Sigma, USA (ref. A3230), respectively.

#### Statistical analysis

All data are represented as mean ± s.e.m. Statistical significance was calculated using a Student’s two-tailed t-test or ANOVA in *Drosophila* WB. If p<0.05 in the ANOVA test, a Bonferroni posthoc t-test was applied. The Kolmogorov-Smirnov test for normality was performed where appropriate, prior to the use of the Student’s two tailed t-test. GraphPad Prism software package versions 3 and 6 (GraphPad, CA, USA) were used throughout. Significant differences were noted by *p<0.05, **p<0.005 and ***p<0.0001.

## Results

### GSK3 modulates synapse number in Drosophila

**Fig 1:**
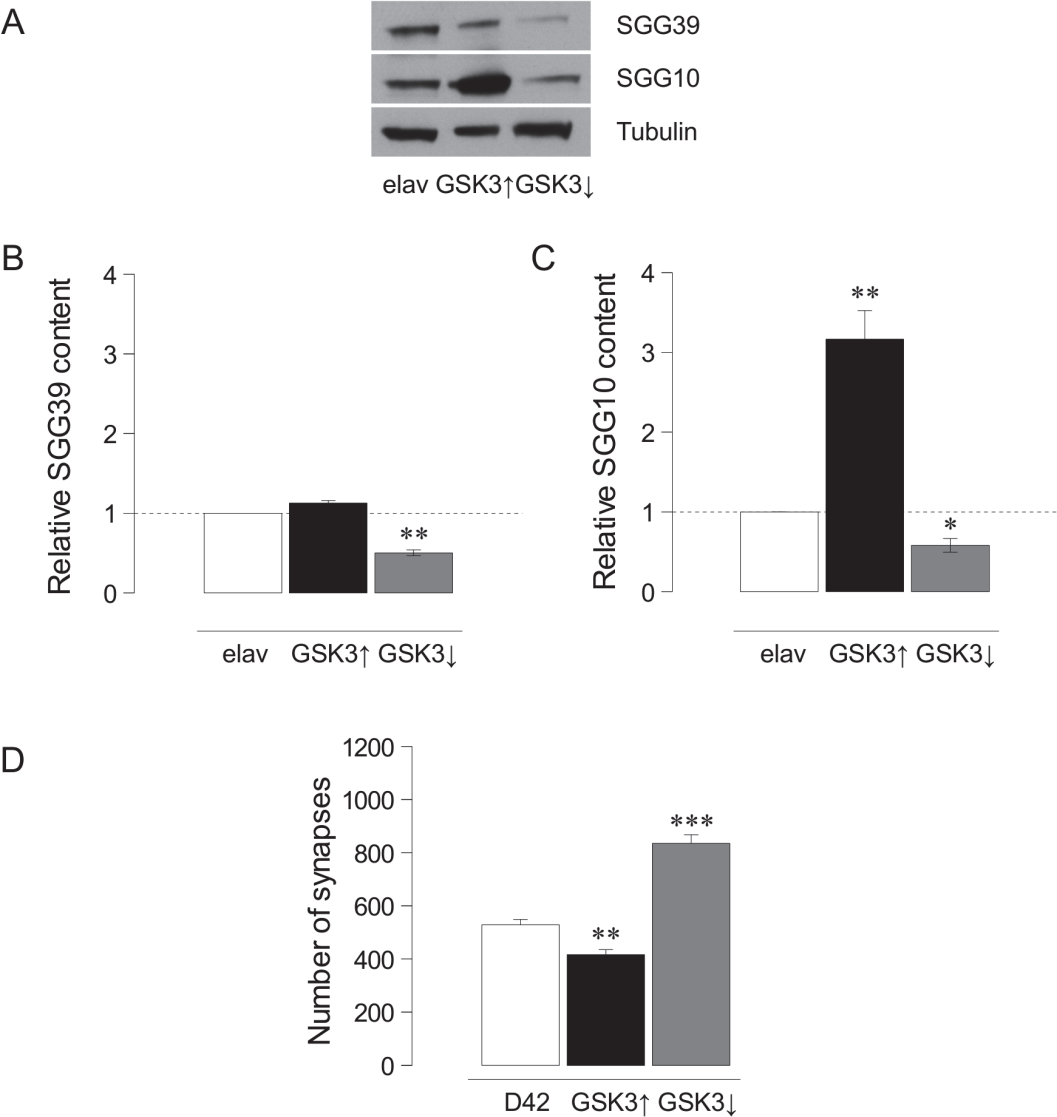
Quantification of GSK3 isoforms levels and synaptic effects. **A.** Image from a representative immunoblot showing GSK3 levels in larval brains. The antibody recognizes the two larval GSK3 isoforms SGG39 (upper band) and SGG10 (middle band). Tubulin is shown as load control (lower band). **B.** Histograms show the content of SGG10 isoform in controls (*elav-Gal4*), GSK3 over-expressers (*elav-Gal4/UAS-GSK3*) and GSK3 under-expressers (*elav-Gal4/UAS-GSK3* ^*RNAi*^) normalized to Tubulin (n = 11 larval brains per lane, n = 3 independent Western blots). **C.** Histograms show the quantification of SGG39 levels in the three genotypes as above. ANOVA test, *p<0.05, **p<0.005. **D.** Histograms indicate the total number of synapses quantified in GSK3, up- or downregulation. Student's t-test, **p<0.005 ***p<0.0001.

We showed previously that the upregulation of *shaggy/GSK3* reduces synapse number [28,31]. Here, we address the effects of down-regulating this gene and, for consistency, revisited the upregulation experiments. To validate the genetic tools to up- (*UAS-GSK3*) or down-regulate (*UAS-GSK3* ^*DN*^ or *UAS-GSK3* ^*RNAi*^) the gene, we first quantified the expression changes of the two protein isoforms in larvae: SGG39 and SGG10 [15]. Western blots from *elav-Gal4*, *elav-Gal4/UAS-GSK3* and *elav-Gal4/UAS-GSK3* ^*RNAi*^ larval central nervous systems (**Fig. 1A**) indicate that the upregulation of GSK3 does not cause a significant increase of isoform SGG39 levels (**Fig. 1B**), but a strong one (316 ± 36%, p<0.005) in the case of SGG10 isoform (**Fig. 1C**) (n = 11 brains per lane in each genotype). In turn, the downregulation by GSK3^RNAi^ decreases the protein content to 50 ± 4% in SGG39 and to 58 ± 8% in SGG10 (p<0.05 and p<0.05 respectively) (**Fig. 1 B-C**). Thus, both genetic tools effectively modify the gene expression.

To monitor the effects on synapse number, we confirmed that GSK3 overexpression reduces synapse number by 22% (*D42-Gal4/UAS-GSK3*: 417±18, n = 13 larvae *vs*. *D42-Gal4*: 528±35, n = 9 larvae; p = 0.0026) (**Fig. 1D**; **S1A Fig., S1B**). Next, we analyzed the downregulation effects: whereas the dominant negative form of GSK3 proved ineffective (not shown), the GSK3^RNAi^ caused a significant (p<0.0001) increase: 835±38 synapses (n = 7 larvae) when compared to control: 528±35 synapses (n = 9) (**Fig. 1D; S1B Fig.)**. Together, these data demonstrate that the alterations of GSK3 levels elicit changes which are coherent with, and opposite to, the direction of changes in the number of synapses.

### GSK3 is functionally downstream from AKT and PI3K in the synaptogenesis pathway

**Fig 2:**
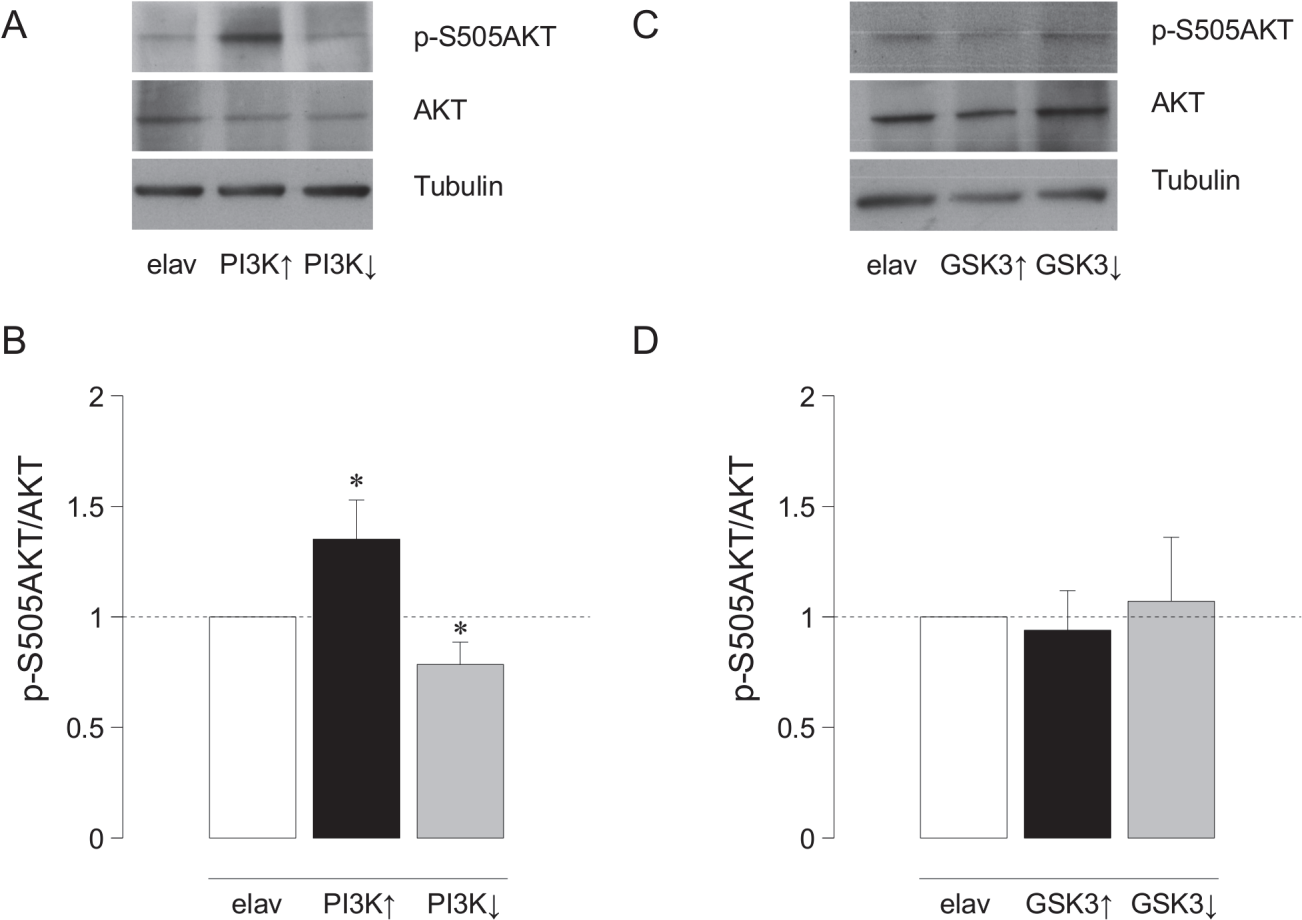
Quantification of p-S505AKT/AKT ratio after up- or downregulation of PI3K or GSK3. **A.** Representative immunoblot showing p-S505AKT and AKT in control (*elav-Gal4*), PI3K over-expressers (*elav-Gal4/UAS-PI3K*) and PI3K under-expressers (*elav-Gal4/UAS-PI3K* ^*DN*^). **B.** Histogram showing the p-S505AKT/AKT ratio in control and experimental larval brains. ANOVA test, * p <0.05. **C.** Representative Western blot showing p-S505AKT and AKT levels in control (*elav-Gal4*), GSK3 over-expressers (*elav-Gal4/UAS-GSK3*) and GSK3 under-expressers (*elav-Gal4/UAS-GSK* ^*RNAi*^) larval brains normalized to Tubulin. **D.** Plot showing the corresponding p-S505AKT/AKT ratio in these genotypes (n = 11 larval brains per lane, n = 4 independent Western blots).

As it is well known in several signaling pathways, PI3K modulates AKT activity. To confirm the case in the *Drosophila* nervous system, we quantified Western blots of genotypes that up- or down-regulate PI3K. The ratio p-S505AKT/AKT increases 35±18% (p<0.05) in brains over-expressing PI3K (*elav-Gal4/UAS-PI3K*; n = 11 brains per lane in each genotype) and decreases 22±10% (p<0.05) in brains over-expressing a dominant negative form of PI3K (*elav-Gal4/UAS-PI3K* ^*DN*^) compared to control (*elav-Gal4*) (**Fig. 2A, 2B**). Thus, PI3K modulates the phosphorylation state and, hence, the activity of AKT.

Next, we addressed the relationship between GSK3 and AKT. *Drosophila* has only one serine/threonine protein kinase encoding gene, *Akt1 (dAkt1)*, homologous to mammalian *c-Akt* [53]. Interestingly, as in vertebrates, *Drosophila* AKT inhibits GSK3 by specific phosphorylation of Serine 9 [54]. We asked whether changes in GSK3 could alter the phosphorylation levels of AKT looking for a putative feedback effect. We quantified the ratio of p-S505AKT/AKT in GSK3 up- or down-regulated genotypes. The data show that AKT phosphorylation is not affected by changes in GSK3 expression (**Fig. 2C-D**). Thus, the synaptic effects of GSK3 manipulations proceed through a feed-forward mechanism that does not involve changes in AKT phosphorylation.

**Fig 3:**
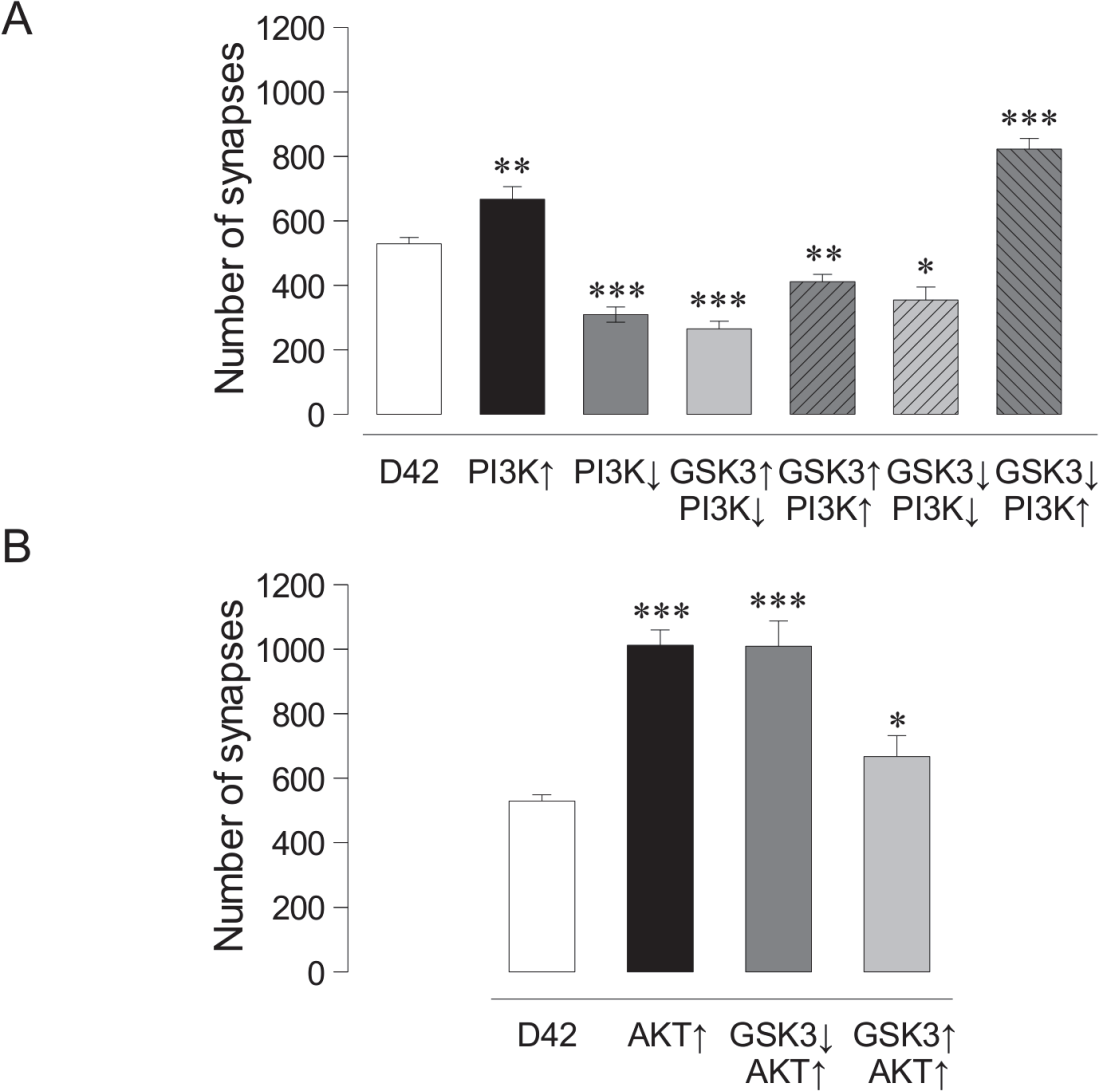
Effects of kinases on larval synapse number. **A.** Histograms indicate the total number of synapses quantified in different GSK3 and PI3K combinations. **B.** Histograms show AKT overexpression and its combination with GSK3 down- and up-expression. Student's t-test, *p<0.05, **p<0.005 ***p<0.0001.

To address the functional relationship *in vivo* between PI3K, AKT and GSK3 in the context of synaptogenesis, we tested their epistatic phenotypes. First, we confirmed the synaptic effects of PI3K to allow a comparison with the subsequent double mutant combinations. As previously reported, PI3K overexpression increases synapses (745±33, n = 8; p<0.0001) and its downregulation decreases them (309±18, n = 8; p<0.0001) compared to control (528±35, n = 9) (**Fig. 3A**).

Next, we assayed combinations of up and down expressions of PI3K and GSK3. The data indicate that the reduction in synapse number elicited by GSK3 overexpression is epistatic over, either the upregulation (*D42-Gal4/UAS-PI3K/UAS-GSK3*, 411±23, n = 8, p<0.005) or the downregulation of PI3K (*D42-Gal4/UAS-PI3K* ^*DN*^/*UAS-GSK3*, 265±24, n = 9, p<0.0001) (**Fig. 3A; S1C–E Fig.**). These results indicate that, following the direct upregulation of GSK3 by genetic procedures, the synaptic effects of this enzyme are independent from any potential regulation from PI3K. By contrast, the increase of synapse number elicited by the downregulation of GSK3 is abolished and reversed when PI3K is down-regulated (*D42-Gal4/UAS-PI3K* ^*DN*^/*UAS-GSK3* ^*RNAi*^, 355±40, n = 7, p<0.05), but maintained when PI3K is over-expressed (*D42-Gal4/UAS-PI3K/UAS-GSK3* ^*RNAi*^; 822±33, n = 7, p<0.0001) (**Fig. 3A; S1D–E Fig.**). Since the genetic downregulation of GSK3 still leaves about 40% expression of both isoforms (**Fig. 1**), these *in vivo* data are compatible with a regulatory role GSK3 upon PI3K activity in the context of synaptogenesis. Thus, akin to other functional contexts, GSK3 and PI3K seem to share the same pathway for synaptogenesis where GSK3 would be a negative modulator of PI3K without evidences of a feedback regulatory loop.

Concerning the epistasia analysis between AKT and GSK3, we first confirmed that AKT upregulation increases synapses (**Fig. 3B** and **S1F Fig.**, *D42-Gal4>UAS-AKT*, 1012±48, n = 8) compared with controls (*D42-Gal4*, 528±35, n = 9) (p<0.0001). The combined AKT overexpression/GSK3 downregulation showed a synaptic increase of the same magnitude as the stronger of the two, AKT (**Fig. 3B** and **S1G Fig.**, *D42-Gal4>UAS-AKT>UAS-GSK3* ^*RNAi*^, 1001±79, n = 9, p<0.0001). Finally, when GSK3 and AKT were simultaneously over-expressed we obtained a modest but significant increase in synapse number (**Fig. 3B**, *D42-Gal4>UAS-AKT>UAS-GSK3*, 667±65, n = 5, p<0.05). GSK3 combinations against a homozygous AKT null background (*D42-Gal4>UAS-GSK3; AKT* ^*–/–*^ or *D42-Gal4>UAS-GSK3* ^*RNAi*^ *; AKT* ^*–/–*^) were not viable and, thus, we limited our study to AKT over expression condition only. Taken together, the data indicate that GSK3 levels do not modify the synaptogenic effects of AKT upregulation, placing GSK3 functionally downstream of AKT in the synaptogenic pathway.

### GSK3 inhibition can be achieved in mammalian neurons

We reasoned that, if GSK3 also participates in the PI3K-synaptogenic pathway of vertebrates, then the GSK3 inhibition might result in a similar effect. We inhibited GSK3 activity through two different approaches: 1) by stimulating its PI3K-AKT-dependent phosphorylation using an engineered peptide, and 2) by its pharmacological inhibition using two high-affinity and selective organic compounds.

**Fig 4:**
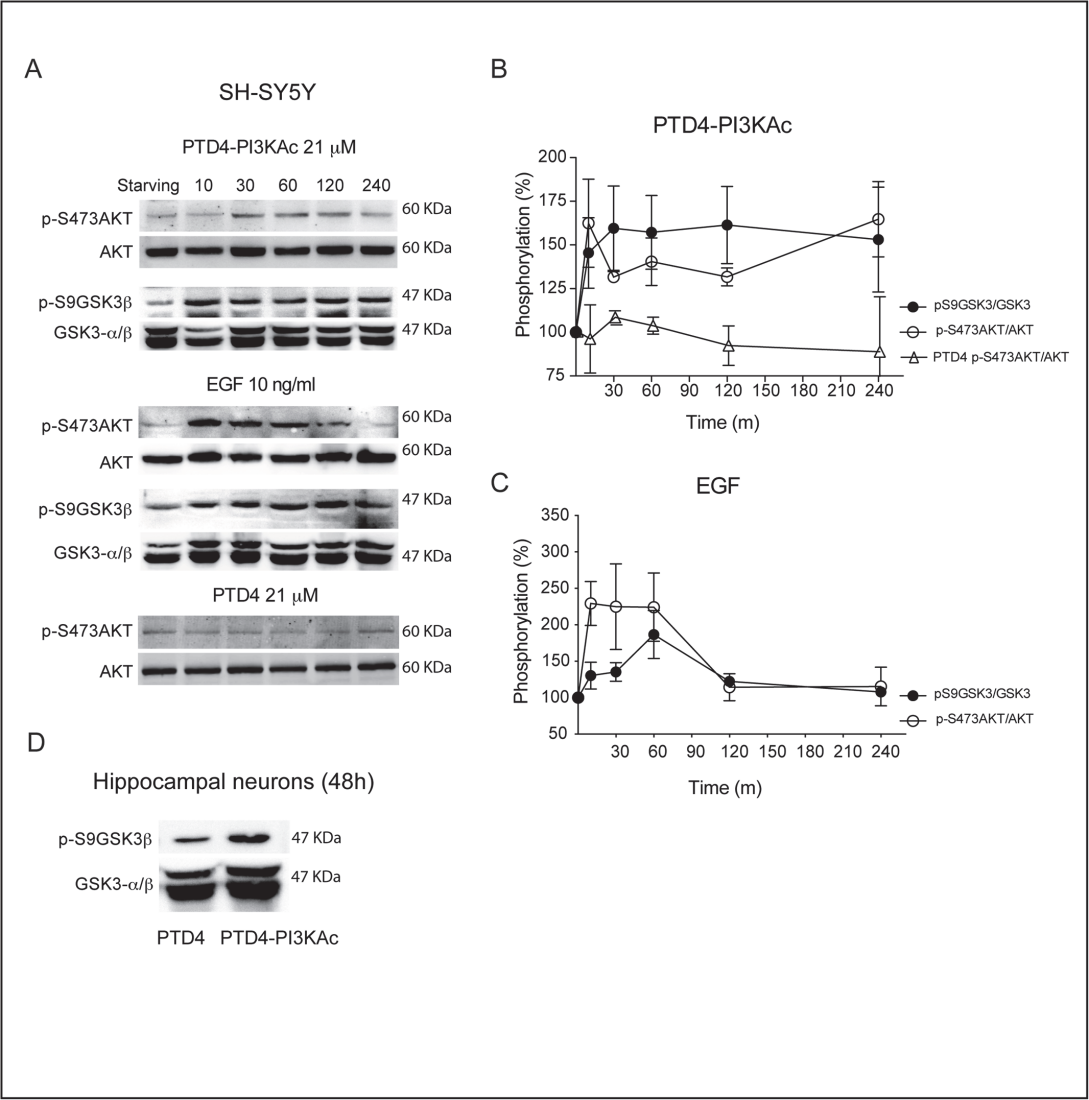
The PI3K activator peptide PTD4-PI3KAc activates AKT and induces phosphorylation of GSK3 at S9. **A.** SH-SY5Y cell cultures were serum starved for 16 hours and challenged afterwards with PTD4-PI3KAc 21 μM, EGF 10 ng/mL or PTD4 (control peptide at a concentration of 21 μM). Cells were lysated at the indicated times and membranes incubated with anti-p-S473AKT and anti-p-S9GSK3β. Levels of phosphorylation were normalized to AKT and GSK3β (lower band), respectively. **B.** Quantitative measurements of p-S473AKT (open circles) and p-S9GSK3β (closed circles) after PTD4-PI3KAc treatment (n = 4). The level of p-S473AKT after PTD4 treatment (open triangles) is included as a negative control (n = 3). **C.** Quantification of p-S473AKT (open circles) and p-S9GSK3β (closed circles) levels after EGF (10 ng/ml) treatment (n = 5). **D.** Hippocampal neuronal cultures of 12 DIV were treated for 48 hours with the PTD4-PI3KAc or PTD4 peptides. The treatment results in an average increase of p-S9GSK3β levels of 121 ± 3% (n = 7 from 7 different cultures). Student’s two-tailed t-test, ***p<0.0001.

To examine if the PI3K activator peptide can modulate GSK3β phosphorylation, we took advantage of a PI3K previously characterized activation peptide (PTD4-PI3KAc; [29]) (see **Material and Methods**) and quantified the levels of p-S473AKT and p-S9GSK3β [10], on SH-SY5Y human neuroblastoma cell line cultures. Due to the presence of fetal serum in the culture media, p-AKT levels are high under normal conditions; thus, to enhance the signal, cells were serum starved for 16 hours prior peptide addition. The activity time course shows an increase of p-S473AKT/AKT levels, reaching a 162±2% after 10 minutes and remaining stable with a value around 140% for the rest of the experiment (240 min; **Fig. 4A**). In parallel, p-S9GSK3β/GSK3β levels peaks after 30 minutes, reaching plateau (157±2%) for the rest of the experiment (**Fig. 4B**).

As a positive control, we employed EGF, a *bona fide* tyrosine kinase activator. EGF induces a rapid and large increase of p-S473AKT/AKT (229±3% after 10 min) that decreases to basal levels (around 100% after 120 min). Levels of p-S9GSK3β/GSK3β show a similar profile with a maximum of phosphorylation after 60 min (186±3%) and a later reduction to basal levels 240 min later (100%) (**Fig. 4C**). As a negative control, cells were treated with the transduction PTD4 peptide without the PI3K activation domain (**Fig. 4B**). Similar results over p-S9GSK3β were obtained with hippocampal neurons in culture: after 48 hours treatment with the PI3K activator peptide, levels of p-S9GSK3β/GSK3β raised to a statistically significant, 127±4% (p<0.0001), over control conditions (**Fig. 4D**). All together, these experiments validate the PTD4-PI3KAc peptide as an effective tool to modulate GSK3 phosphorylation, and point out to an important feature of the peptide: although the activation level of AKT is weaker than that of a tyrosine kinase activator (EGF), its biological activity remains stable for a longer period of time, up to 48 hours. Further, the data demonstrate that the activation of AKT by the peptide results in GSK3β phosphorylation at Ser 9.

To pharmacologically block GSK3β, we used two specific organic inhibitors: AR-A014418 (AR) [50,55] and SB 415286 (SB) [51–52, 56] with an in vitro IC50 of 0.104 and 0.13 μM, respectively. Prior to use them, we studied their potential toxicity over hippocampal neurons in culture. Cell survival was quantified 48 hours after addition of different concentrations of both drugs (**S2A Fig.**). SB did not affect neuronal density at a concentration range of 10 to 25 μM, but the use of 50 μM resulted on a not statistically significant reduction of 13.8% on neuronal survival (**S2B Fig.**). The second inhibitor, AR, did not affect neuronal survival at 1 and 10 μM, while at 50 μM induced a significant neuronal death of 16.5% (p<0.05) (**S2C Fig.**).

**Fig 5:**
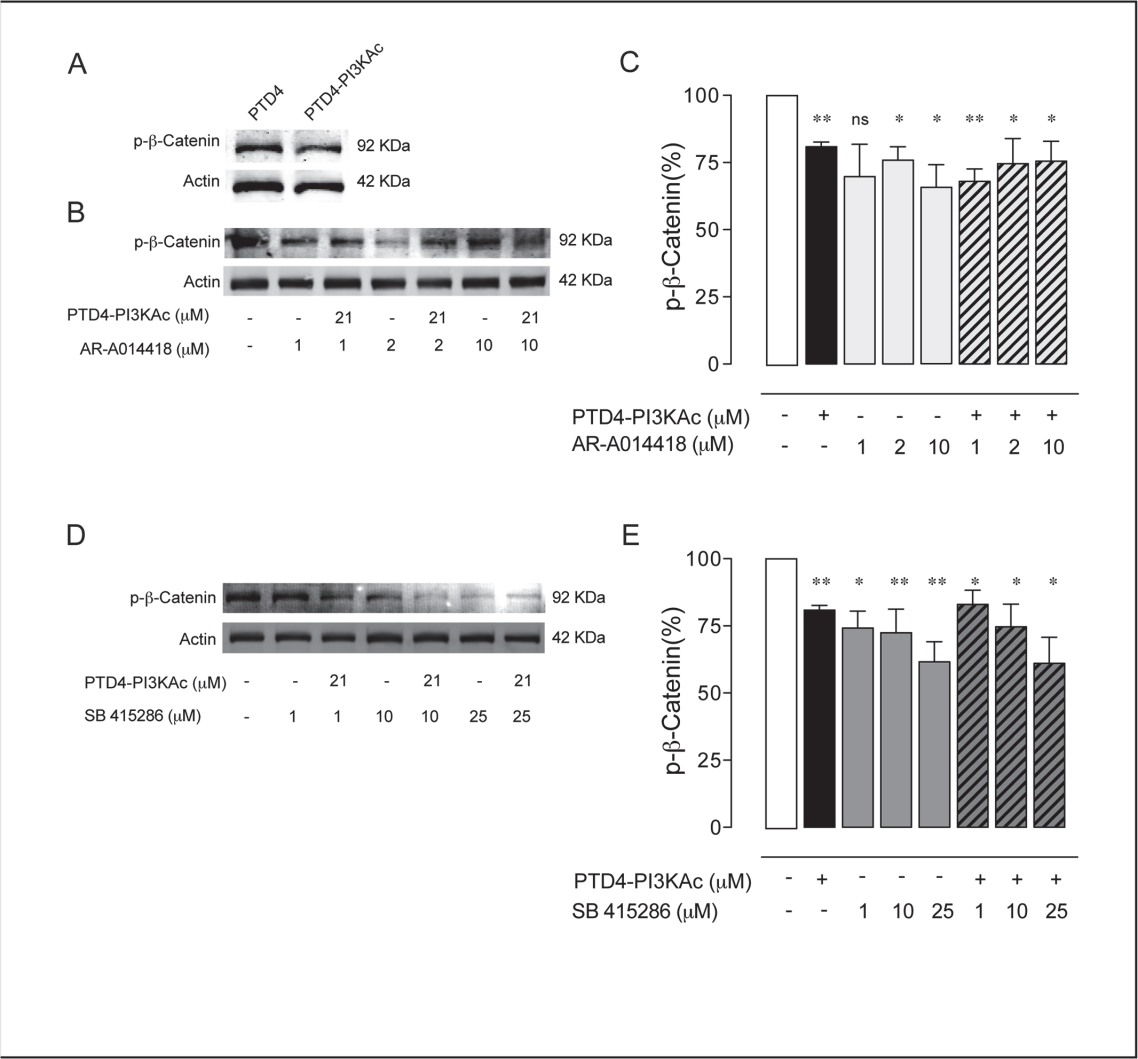
GSK3 inhibition results in a decrease of p-β-Catenin. **A.** Immunoblot of p-β-Catenin from 12 DIV hippocampal neurons treated for 48 hours with PTD4-PI3KAc or PTD4 (21 μM). **B.** Immunoblot of p-β-Catenin after AR-A014418 treatment, alone or in combination with PTD4-PI3KAc (21 μM). Phosphorylation levels were normalized to the total actin. **C.** Plot bar representing the mean average of p-β-Catenin after the described treatment. All percentages are referred to control conditions as 100%, always included in each Western-blot membrane. Only the treatment with 2 and 10 μM of AR-A014418 decreased p-β-Catenin levels. The co-treatment with the PI3K activator did not induce a further reduction. **D.** Immunoblot of p-β-Catenin after SB 415286 or in combination with PTD4-PI3KAc (21 μM) treatments. **E.** Plot bar of the percentage of p-β-Catenin after treatment with SB 415286. Exposure of cells to SB 415286 or in combination with PI3K activator led to a statistically significant decrease in p-β-Catenin (n = 15 from 15 different cultures). Percentage of p-β-Catenin was normalized to the total Actin levels. Significance refers to the statistical analysis of the experiments versus the control (100%). Student’s two-tailed t-test, *p<0.05, **p<0.005.

We next assayed the effectiveness of these drugs to inhibit GSK3 in hippocampal neurons in culture. GSK3 regulate β-Catenin levels by phosphorylation at both Serine 33 and 37 and also at Threonine 41 [57]. Since both organic compounds block the ATP pocket of all GSK3 isoforms [51], we monitored the relative levels of β-Catenin phosphorylation as a reporter of GSK3 activity [36,58]. First, the ability of PTD4-PI3KAc to regulate phospho-β-Catenin levels was tested. In neuronal cultures a treatment of 21 μM PTD4-PI3KAc for 48 hours reduced p-β-Catenin levels by 19±2% (p<0.005) (**Fig. 5A, 5C**). The effects of AR were tested at 1, 2, and 10 μM (**Fig. 5B**). No significant changes were observed at 1μM; while 2 μM and 10 μM reduced it 24±5% and 35±8%, respectively (p<0.05 in both experiments) (**Fig. 5C**). The simultaneous activation of PI3K by PTD4-PI3KAc and the direct inhibition of GSK3 with AR did not result in a further inhibition of GSK3β pathway, with an average reduction of 32±5% (p<0.005), 25±9% (p<0.05), and 24±7% (p<0.05) at 1, 2 and 10 μM of AR respectively, suggesting that both drugs act over the same pathway (**Fig. 5C**). The efficiency of SB inhibiting GSK3β was tested at 1, 10 and 25 μM, the levels of p-β-Catenin decreased in a 26±6% (p<0.05), 28±9% (p<0.005) and 38±8% (p<0.005) respectively (**Fig. 5D, 5E)**. Similar to AR, co-treatment of PTD4-PI3KAc and SB did not further decrease the phosphorylation levels of β-Catenin (17±5% (p<0.05), 25±8% (p<0.05) and 39±10% (p<0.05) at 1, 10 and 25 μM of SB, respectively; **Fig. 5E**). In conclusion, PTD4-PI3KAc and the inhibitors AR and SB effectively inhibit GSK3β at the selected dose.

### GSK3 inhibition exhibits an age-dependent effect on mammalian synaptogenesis

**Fig 6:**
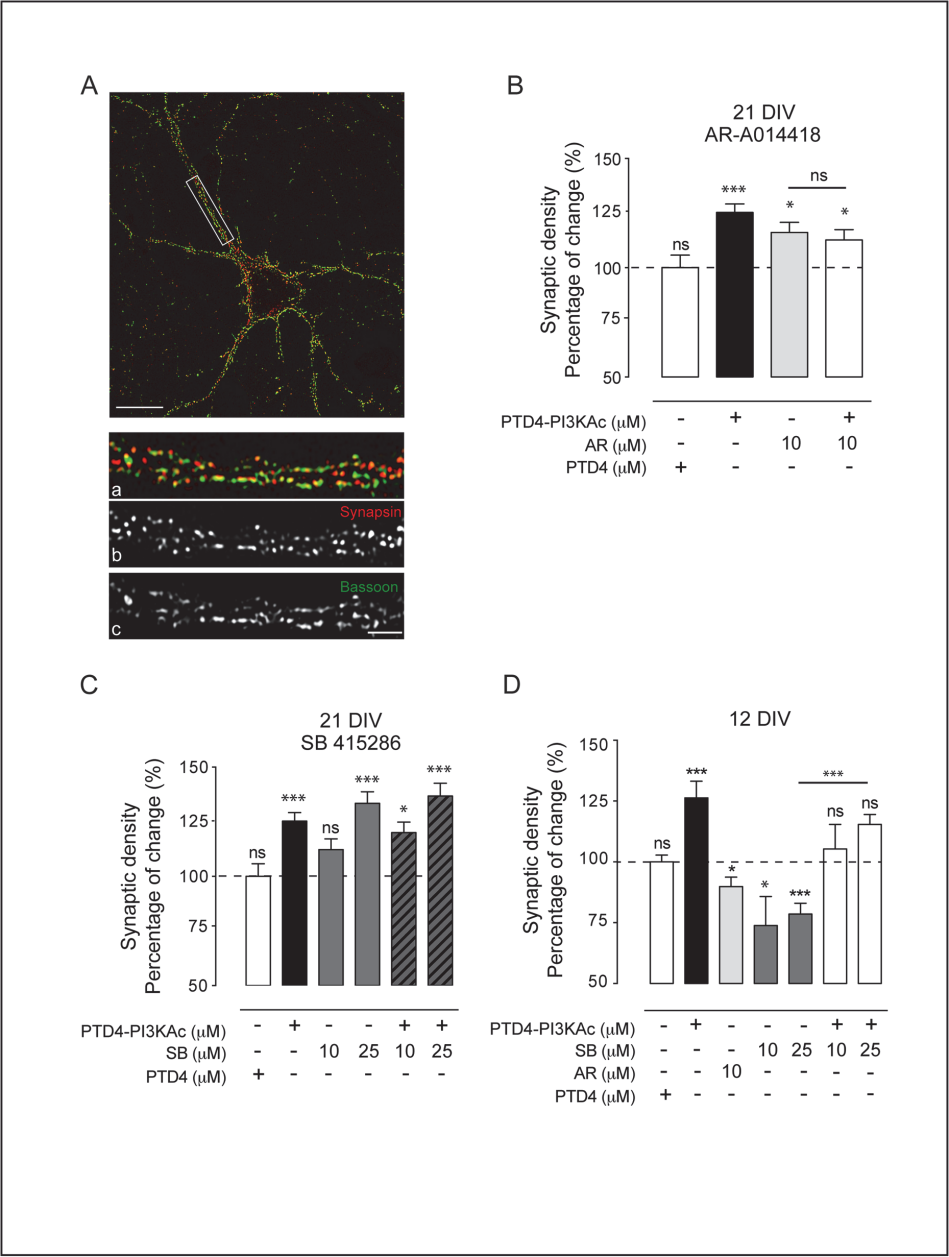
Differential effects of GSK3 inhibition on synaptic density. **A.** Example of a 21 DIV hippocampal neuron in culture. Scale bar = 10 μm. Bottom pictures: detailed magnification of the area depicted by the white box. Synapses (a) were localized following a double criterion: puncta positive for Synapsin (b, red) and for Bassoon (c, green). Scale bar 3 μm. **B.** Plot graph of the average changes in synaptic density at 21 DIV after AR-A014418 treatment. PTD4-PI3KAc and PTD4 were used at 21 μM. **C.** Plot graph of the results of synaptic density after the GSK3 inhibition by SB 415286 treatment. **D.** Quantification of synaptic density at 12 DIV after treatments with PTD4-PI3KAc, AR-A014418, SB 415286 alone or in combination with PTD4-PI3KAc. Percentage of change refers to PTD4 control treatment. Cultures were treated for 48 hours before fixation (n = 10–12 from 3 different cultures). Significance refers to the statistical analysis of each experimental condition versus PTD4 treated cultures (100%) Student’s two-tailed t-test, *p<0.05, **p<0.005, ***p<0.0001.

The density of synapses was evaluated by immunocytochemistry on hippocampal cultures (**Fig. 6A**). The overlap of two presynaptic markers (Bassoon, a scaffold protein, and Synapsin, a vesicular component) was used as criteria for mature synapses as previously described [29,59]. We employed fully developed cultures of 21 DIV, applying the treatment at day 19 and counting synapses at day 21. At this age, PTD4-PI3KAc (21 μM) induced an augmentation of synaptic density (**Fig. 6B, 6C**) to 125±4% (statistically significant, p<0.0001). This observation is in agreement with our previous data showing an increase in both excitatory and inhibitory synapses following PI3K up-regulation [29]. Conversely, GSK3β inhibition by AR 10 μM increased synaptic density to 116±v5% (p<0.05) (**Fig. 6B**) and SB raised synaptic density to 112±5% (ns) and 133±5% (p<0.0001) at 10 and 25 μM respectively (**Fig. 6C**). To investigate potential cooperative effects, we analyzed the simultaneous activation of PI3K and inhibition of GSK3. The combined treatment of PTD4-PI3KAc with AR results in an increase of 113±5%, ns, while the use of SB resulted in synapse density raises of 120±5% (p<0.05) and 137±6% (p<0.0001), 10 and 25 μM respectively. In these three cases, the increase was similar to the individual treatments (**Fig. 6B, 6C**); suggesting that GSK3 should be downstream from, and in the same pathway of PI3K in the context of synaptogenesis.

In younger (12 DIV) cultures, PI3K activation resulted also in the expected synaptic increase, 126±6% (p<0.0001) (**Fig. 6D**). However, the inhibition of GSK3 by AR or SB reduced synapse number instead as observed in older cultures. SB at 10 and 25 μM reduced synaptic density to 74±12% (p<0.05) and 79±4%, (p<0.0001) respectively (**Fig. 6D**), and AR reduced it to similar values (90±4%; p<0.05) (**Fig. 6D**). As it seems, the strong pro-synaptogenic effect elicited by GSK3 inhibition in 21 DIV cultures (see 25 μM SB treatment in **Fig. 6C**), is opposite to the effect obtained with the same drug and dosage in 12 DIV cultures (**Fig. 6D**). To assess the independence of PI3K and GSK3 in the process, we measured synaptic density after a combined treatment with SB and PTD4-PI3KAc. The results indicate that activation of PI3K compensates the reduction of synaptic density caused by GSK3 inhibition (statistically significant, p<0.0001 when compared with SB alone), yielding mean values of 105±10 and 115±4% (10 and 25 μM of SB respectively), which are not significantly different from culture controls conditions (**Fig. 6D**). Thus, PI3K is not involved in the age-dependent effect caused by GSK3 inhibition.

**Fig 7:**
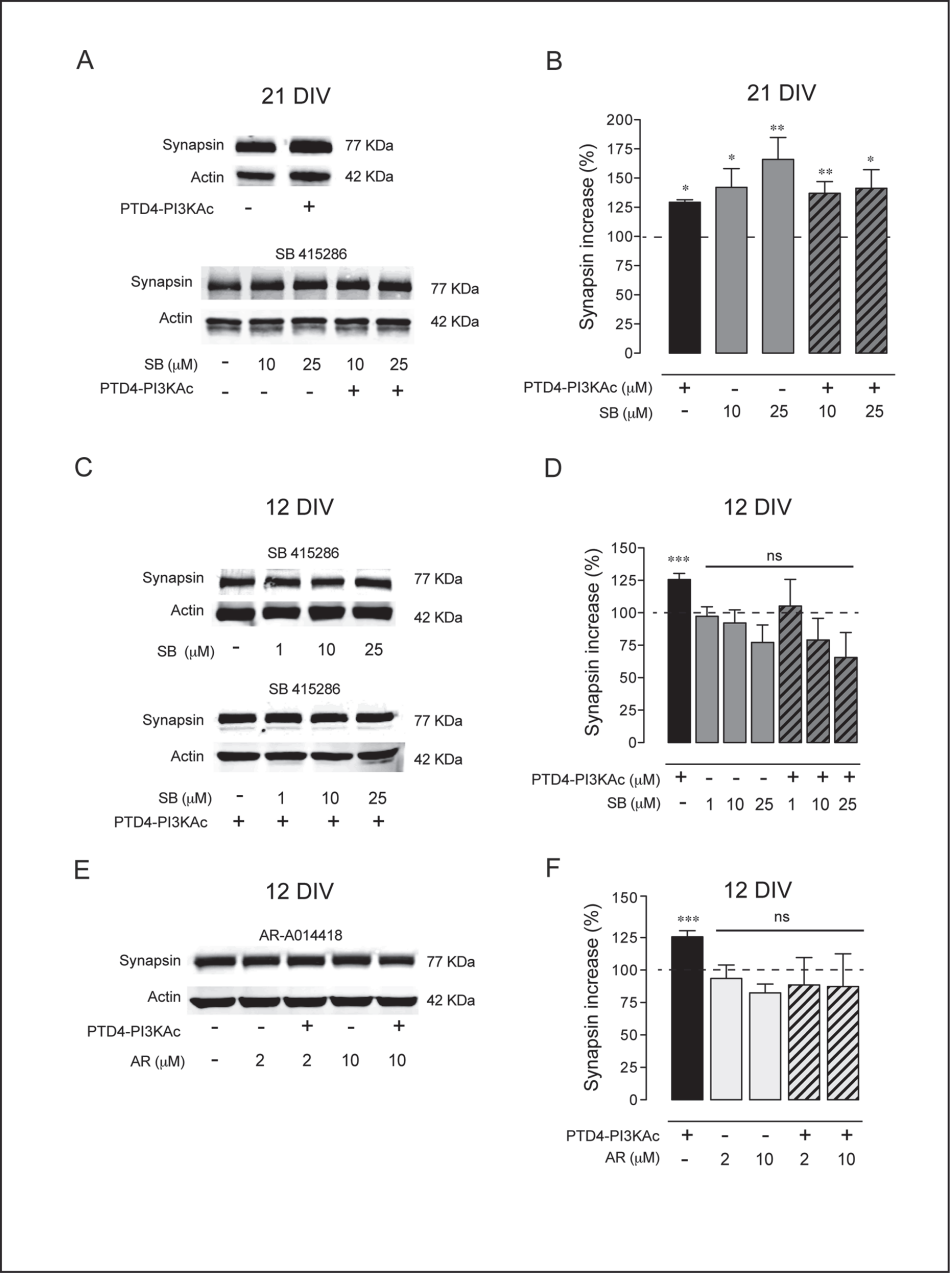
GSK3 inhibition modifies Synapsin levels. **A.** Representative immunoblot showing Synapsin levels after GSK3 inhibition by SB 415286 treatment in 21 DIV neuronal hippocampal cultures. **B.** Plot graph summary of the results of 21 DIV cultures. The bars are the average levels of Synapsin versus control conditions after the indicated treatments, alone or in combination with PTD4-PI3KAc (21 μM). **C.** Representative immunoblot for Synapsin after GSK3 inhibition in 12 DIV cultures **D.** Plot graph of Synapsin after SB 415286 treatment, alone or in combination with PTD4-PI3KAc (21 μM). **E.** Representative immunoblot of Synapsin after GSK3 inhibition by AR-A014418 in 12 DIV cultures. **F.** Plot bars of the average levels of Synapsin after AR-A014418 treatment, alone or in combination with PTD4-PI3KAc (21 μM). Percentage of Synapsin was normalized to the total Actin levels. n = 10–15 from 6 different cultures. Significance refers to the statistical analysis of the experiments versus the control (100%). Student’s two-tailed t-test, *p<0.05, **p<0.005, ***p<0.0001.

To analyze the age-dependent effect of GSK3 inhibition through an alternative method, we quantified the levels of two synaptic proteins, Synapsin and PSD95, by Western blot. Synapsin is the major component of the presynaptic vesicles and its expression has been used as a reporter of synaptogenic activity [29]. Protein levels were quantified from high-density cultures treated during 48 h, either with inhibitors alone or in combination with the PI3K activating peptide. In 21 DIV cultures, activation of PI3K up-regulates Synapsin by 129±2% (p<0.05). Moreover, GSK3 inhibition by SB, the most robust inhibitor, raised Synapsin to 142±2% and 166±2% (10 and 25 μM of SB, p<0.05 and p<0.005 respectively; **Fig. 7A, 7B**). The simultaneous activation of PI3K and inhibition of GSK3 resulted in a similar degree of augmentation, with mean values of 137±1% (p<0.005) and 141±2% (p<0.05) (**Fig. 7B**). These changes in Synapsin levels are consistent with the variation in synapse density (see above, **Fig. 6D**).

In 12 DIV cultures, PI3K activation increased Synapsin by 126±4%, the same effect as in the aged cultures (see above). By contrast, GSK3 inhibition by SB (1, 10 and 25 μM) did not result in a significant change of Synapsin levels (**Fig. 7C, 7D**) as it was observed in the aged cultures (**Fig. 7B**). Similar to 21 DIV, the simultaneous PI3K activation by the peptide and GSK3 inhibition by SB, at all concentrations tested, did not elevate Synapsin in young cultures (**Fig. 7D**). These effects on Synapsin expression were reproduced with AR (**Fig. 7E, 7F**). In this case, neither the 2 or 10 μM AR treatments, nor their combination with PTD4-PI3KAc, modified Synapsin levels (**Fig. 7F**).

**Fig 8:**
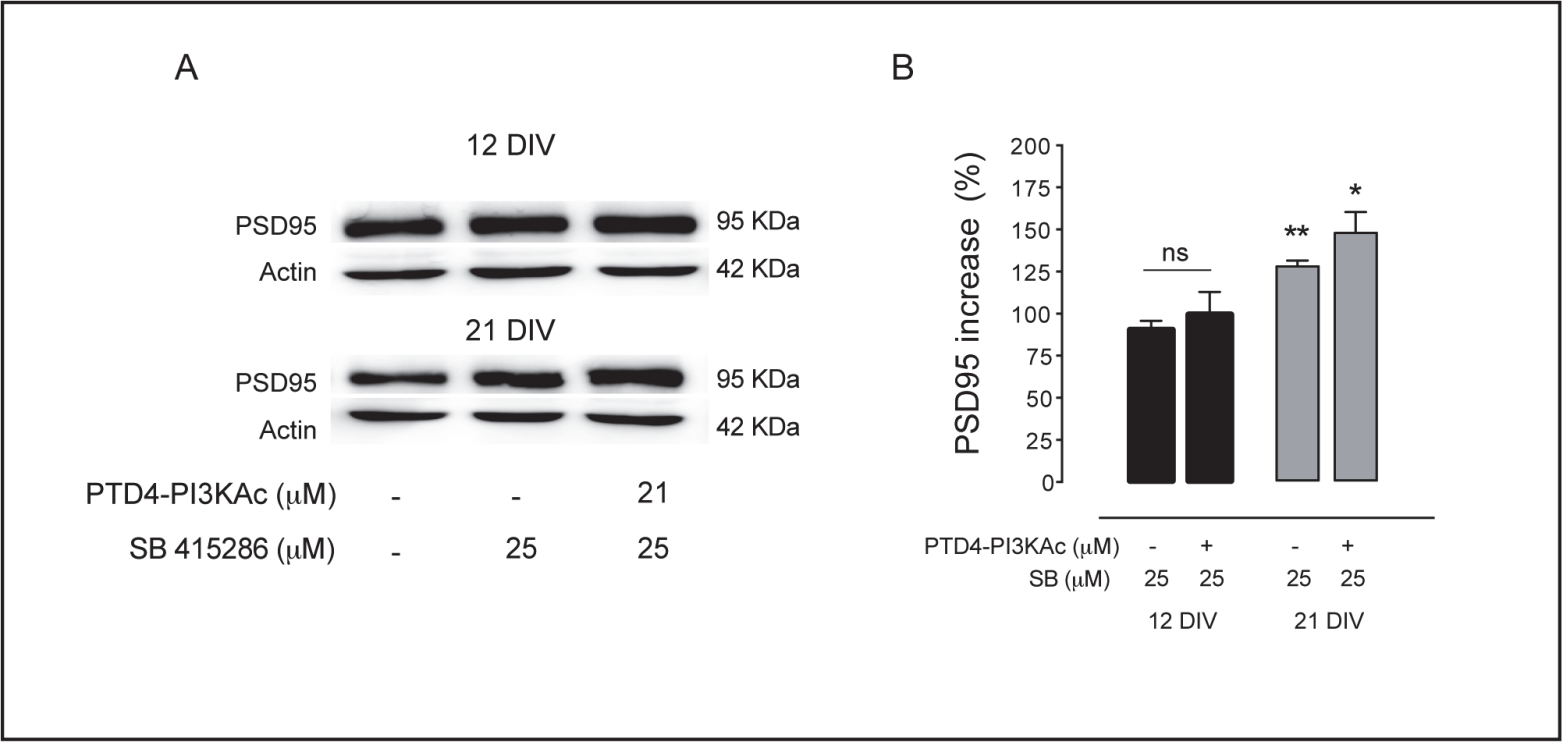
GSK3 inhibition affects PSD95 expression. **A.** Representative immunoblots of PSD95 levels after GSK3 inhibition by SB 415286 treatment employing 12 or 21 DIV cultures. **B.** Plot graph summary of the results of 12 and 21 DIV cultures. The bars are the average levels of PSD95 versus control conditions after SB 415286 treatments, alone or in combination with PTD4-PI3KAc (21 μM). Percentage of PSD95 was normalized to the total Actin. n = 4–5 from 3 different cultures. Significance refers to the statistical analysis of the experiments versus the control (100%) *p<0.05, **p<0.005, ***p<0.0001, Student’s two-tailed t-test.

In addition, we tested another synaptic protein, PSD95, which is a component of the glutamatergic postsynaptic side and obtained similar effects (**Fig. 8**). In 21 DIV cultures GSK3 inhibition by SB raised expression levels to a 128±3% (p<0.005) and the joint activation of PI3K plus the inhibition of GSK3 resulted also in a significant increase of 148±1% (p<0.05). By contrast, the same treatments applied in 12 DIV cultures did not result in significant changes in PSD95 (90±5%, with SB, and 99±1% in the PTD-PI3KAc + SB treatments), (**Fig. 8A, 8B**). Therefore, we conclude that GSK3 inhibition has a differential effect repressing/enhancing synapse formation depending on neuron age and this effect is not related to PI3K.

### GSK3 regulates spine formation

Dendritic spines are postsynaptic specializations whose number has been associated with learning and memory processes [60]. The increase in synaptic density found in 21 DIV cultures prompted us to consider whether spine density was also up-regulated by GSK3 inhibition. GSK3 has been located in spine densities associated to AMPA receptors, location compatible with a role regulating synaptic plasticity and spine formation [19].

**Fig 9:**
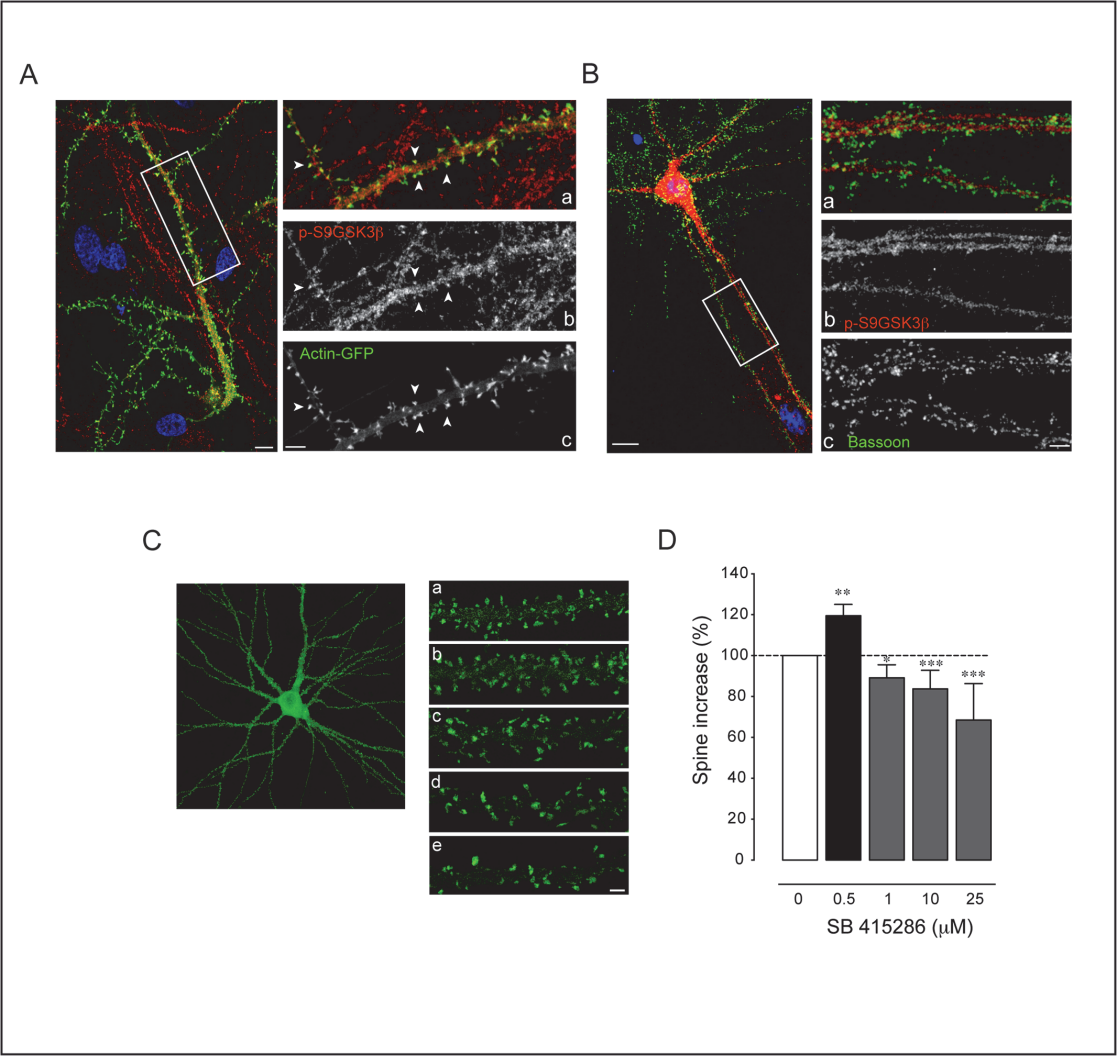
Localization of p-S9GSK3β at the synapse and spine effects of its inhibition. **A. p-S9GSK3β localizes at the postsynaptic side. Left:** Single confocal microscopy image of a GFP-Actin transfected neuron counterstained with p-S9GSK3β in red and nuclei in blue (DAPI). Note that the cell body of the transfected neuron is not in the same focal plane (bottom right corner of the image). Scale bar = 5 μm. **Right:** High magnification view of the area depicted in the white box showing the (a) Merged picture of (b) p-S9GSK3β in red and (c) Actin-GFP signals in green. Positive spines for p-S9GSK3β are indicated by white arrowheads. Scale bars = 3 μm. **B. p-S9GSK3β does not colocalize with presynaptic Bassoon. Left:** 21 DIV old hippocampal neuron stained with p-S9GSK3β in red, Bassoon in green and nuclei in blue (DAPI). Scale bar = 5 μm. Right panels: high magnification view of the area depicted in the white box showing (a) Merged picture of (b) p-S9GSK3β in red and (c) Bassoon in green. Notice the total absence of colocalization between both markers. Scale bar = 3 μm. **C. GSK3 inhibition has a differential effect on spine density.** Actin-GFP transfected neurons of 19 DIV were treated for 48 hours with different concentrations of SB 415286. **a, b, c, d, e,** Dendritic fragments of a control (top), 0.5 μM, 1 μM, 10 μM and 25 μM of SB 415286 treated neurons, respectively. **D.** Plot graph summary of the effects on spine density [number of cultures: n = 3 (0.5 μM); n = 4 (1 μM); n = 3 (10 μM); n = 2 (25 μM)]. Average number of spines were compared and normalized in front of control cultures. Student’s two-tailed t-test, *p<0.05, **p<0.005, ***p<0.0001.

We first examined the localization of p-S9GSK3β in hippocampus neurons transfected with GFP-Actin [61]. The expression vector was under control of a PDGF neuronal promoter to avoid excessive protein expression [45]. After 19 DIV, the GFP-Actin signal was concentrated at the spine domains that facilitate their identification. Under these conditions, p-S9GSK3β was found in dendrites and in most spines (**Fig. 9A**). To discriminate if the protein could localize also in the presynaptic compartment we co-immunolocalized Bassoon. The images indicate that p-S9GSK3β is not associated with the presynaptic side (**Fig. 9B**).

To quantify the effects of GSK3 inhibition over spine density, culture neurons were treated with a broad range of SB concentrations from 0.5 to 25 μM for 48 hours (**Fig. 9C**; [62]). Similar to the case of culture age (see above) GSK3 inhibition by SB has a differential effect over spine density, albeit now dependent on dosage. Thus, 1 to 25 μM SB, produced a dosage-dependent reduction in spine density ranging from 84±9 (p<0.05) to 68±2% (p<0.0001) (**Fig. 9D**). Interestingly, when cultures were treated with the lowest concentration (0.5 μM) spine density exhibit an opposite effect, a significant increment of 16±5% (p<0.005) (**Fig. 9D**). Likely, age and dosage are just two among the several factors that may account for the diversity of synaptic responses to GSK3 inhibition.

## Discussion

We have addressed the role of GSK3 in synaptogenesis and spinogenesis in a comparative study between the neuromuscular junction of *Drosophila* larvae and cultured hippocampal rat neurons. The data indicate that GSK3 inhibition in both species leads to a synaptic increase through a PI3K signaling pathway, in which GSK3 represses synapse formation.

In flies, synapse number increases when GSK3 is down-regulated or when PI3K is up-regulated. In the epistasis analysis, same phenotype combinations are not additive which strongly suggests that both kinases share the same pathway. However, these data do not inform about their hierarchical order. Opposite phenotype combinations could have solved the functional hierarchy. One of these combinations—joint upregulation of PI3K and GSK3 -, produced the phenotype of GSK3 alone, while the other combination—joint downregulation of PI3K and GSK3-, yielded the phenotype of PI3K alone (**Figs. 1D** and **3A**). Both results could be explained taking into account the respective amounts of both proteins. Thus, while GSK3 overexpression increases three fold the amount of Sgg10 isoform (**Fig. 1B**), PI3K upregulation increases p-S505AKT by 35% only **(Fig. 3B)**. This excess of GSK3 might not be effectively inhibited by a lesser amount of p-S505AKT, with the final result of a decrease in synapse number due to the GSK3-dependent antisynaptogenic effect. In the second combination, the joint downregulation of PI3K and GSK3, the PI3K phenotype prevails. This result can also be explained through the relative protein levels elicited by the genetic tools. The RNAi against GSK3 yields a reduction of 50% in the total amount of both GSK3 isoforms, Sgg10 and Sgg39 (**Fig. 1B-C**). By contrast, PI3K^DN^ reduces pS505-AKT around 22% (**Fig. 3B**). In this case, the amount of p-S505AKT could not be enough to phosphorylate, hence inhibit, all the GSK3 protein, leaving a sufficiently activated level of GSK3 to finally cause a reduction in the number of synapses. Additionally, we cannot formally rule out the possible differences between the use of dominant negative and RNAi constructs to reduce expression levels of these kinases, taking into account that, whereas the first interferes with protein function, the second attenuates protein transcription. To assess this variability in the available genetic tools, we do not observe changes in synapse number when we use GSK3^DN^ but we observe satellite boutons as previously described ([32], data not shown). Moreover, when AKT overexpression is combined with GSK3^RNAi^ we do not find an additive effect (**Fig. 2C**) in agreement with the previous analysis of PI3K and GSK3 (**Fig. 2B**).

The three kinases, PI3K, AKT and GSK3 seem to share the same pathway for synaptogenesis because all their same phenotype combinations do not show additive effects (**Fig. 3A, 3B**). Also, the p-S505AKT/AKT ratio does not change in larval brains that over- or down-regulate GSK3 (**Fig. 2D**), suggesting again that GSK3 is downstream from AKT and does not affect AKT phosphorylation. This scenario from *Drosophila* neurons is consistent with evidences from other cell types and functional contexts [36].

In vertebrates, GSK3 has been implicated in several neuronal functions [11] and diseases [36]. Referring to pathological conditions, the high levels of GSK3 in Alzheimer disease and the cognitive deficits that these patients show can be interpreted in the light of the reduction of synapse number that flies and rodents exhibit under genetic or pharmacological conditions. However, could a molecular mechanism be proposed to link GSK3 signaling and synapse loss? In mammals, the Wnt/Dvl/GSK3 pathway is implicated in synapse formation [34]. GSK3 α and β are canonical components of this pathway; Wnt binds to Frizzled-LRP5/LRP6 coreceptors causing Dvl activation and displacing GSK3β from its complex with APC, Axin and β-Catenin. This allows β-Catenin targeting to the nucleus where it regulates gene transcription [63–64]. In the absence of Wnt, GSK3β represses this cascade, leading β-Catenin to degradation [36]. Supporting the role of Wnt-GSK3 on synaptogenesis, neurons over-expressing Dvl present a large number of Synapsin clusters in cerebellum, whereas hippocampal neurons treated with soluble Wnt7b show an increase in Bassoon presynaptic clusters [23]. GSK3 inhibition by lithium mimics Wnt7a signaling by inducing axonal remodeling and Synapsin clustering [7]. Furthermore, *in vivo* studies have demonstrated that structural plasticity changes induced by enriched environment are regulated through Wnt7a/b [65]. Also, Wnt7 injection in the hippocampus increases the number of synaptic terminals, whereas perfusion of the Frizzled receptor antagonist, s-FRP1, blocks the effects of an enriched environment over structural plasticity and reduces synapse number [65]. To date, however, no studies had been carried out to analyze the effects of directly inhibiting GSK3 over synaptic density. Here, we regulated GSK3β activity by two different procedures, indirectly, employing a PI3K activator peptide, through the PI3K-AKT-mediated phosphorylation of GSK3 at Serine 9 [66], and directly by blocking kinase GSK3 activity with two chemical inhibitors.

In mature cultures (21 DIV), PI3K activation or direct GSK3 kinase inhibition induced an increase of synapse number, concomitantly with an increase of Synapsin and PSD95 expression. The lack of an additive effect of the conjoint activation of PI3K and GSK3 inhibition, suggest that both kinases share the same pathway. Results in both species confirm a direct role of GSK3 regulating synapse formation and suggest that, under physiological conditions, GSK3 activity represses synapse formation. Inhibition of GSK3 up-regulates PSD95 or Synapsin indifferently, suggesting that GSK3 could operate downstream from PI3K/AKT in the pathway that leads to synapse formation.

When the same experimental procedure was performed in 12 DIV hippocampal neurons, inhibition of GSK3 by PI3K-AKT activation led to an increase of PSD95 and Synapsin expression and a concomitant raise in synaptic density. In contrast, blocking GSK3 kinase activity induced a reduction of synaptic density without affecting Synapsin or PSD95 levels.

Concerning spine density, it has been previously demonstrated that PI3K activation increases spinogenesis both in cultures and in brain hippocampus [29,61,67]. Here, whereas the GSK3 inhibitors raised synaptic density in older cultures, the same range of concentrations reduced spine density and only the lowest concentration up-regulated spine density. Similar results over spine density were reported previously using a related inhibitor of GSK3 [68]. Thus, neurons treated with oligomeric Aβ peptides showed a pronounced spine loss, while the pharmacological inhibition of GSK3 rescued spine loss. Yet, excessive inhibition of GSK3 resulted again in a reduction of spine number [55]. On the mechanism of spine loss by GSK3 inhibition, at this point we can only speculate with an interaction of postsynaptic GSK3 with actin polymerization [69]. Further experiments analyzing actin polymerization in spines will be required to clarify this point. It is plausible, however, that synaptogenesis and spinogenesis will be sustained by non-identical mechanisms.

Why GSK3 inhibition can repress/enhance synaptic formation depending on the age of the cultured neurons? To answer this question we must consider that GSK3 regulation can be independent from AKT phosphorylation. Indeed, downstream of PI3K, GSK3 is inactivated via AKT-mediated phosphorylation of two serine residues (serine 21 for GSK3α and serine 9 of GSK3β; [36]). When both native α and β GSK3 are replaced by mutant isoforms resistant to inhibitory PI3K/AKT phosphorylation, the resulting double GSK3 knock-in mice develop normally with no defects in their nervous system [70] and cultured hippocampal neurons polarize normally [55]. However, blocking GSK3 by isoform-specific inhibitors alters neuron polarization and induces formation of multiple axons [7]. Recently, Gobrecht and colleagues have demonstrated that GSK3 knock-in mice show an accelerated axonal growth of Dorsal Root Ganglion (DRG) neurons both in culture and *in vivo*, an effect that was impaired by the use of a direct GSK3 kinase inhibitor (i.e. SB) [56]. Furthermore, another recent work on DRG sensory axons showed that regenerative properties do not depend of GSK3 phosphorylation. Interestingly, the inhibition of PI3K in these neurons results in a potentiation of GSK3 kinase activity, pointing out the existence of an alternative PI3K-dependent pathway to inhibit GSK3 [71]. All together, these findings suggest that AKT-mediated phosphorylation might not be the only mechanism by which GSK3 is inactivated in neurons downstream of PI3K. The levels of GSK3 expression, rather than its phosphorylation status, might determine its activity in neurons.

Alternatively, a possible change in Wnt signaling along hippocampal cell culture development could also be responsible for the observed differential GSK3 effects. Wnt and PI3K-AKT pathways share a common element, Axin, which binds to GSK3 preventing its phosphorylation and the subsequent inhibition by AKT [72]. Inhibitors binding to the catalytic pocket of GSK3 could regulate GSK3 independently of Axin function and AKT phosphorylation [73]. Developmental changes either in the level of GSK3 expression or apparent affinity for the Wnt-Axin complex should modulate GSK3 activity. Future experiments characterizing the synaptogenic PI3K role in the double GSK3 knock-in mice would be helpful to clarify this point.

Other authors have also proposed an additional layer of GSK3 regulation based on the existence of “primed” or “not primed” substrates. The prior phosphorylation of many GSK3 targets enhances GSK3 affinity and binding to such primed substrates [52,74–75]. Thus, a developmentally regulated expression of GSK3-phosphorylated substrates may explain the differential effects of the chemical inhibitors. It is worth noting, however, that PI3K activation exerts a synaptogenic role independently of culture age ([29], this paper).

In summary, this study shows that GSK3β regulates synapse number in flies and rodents through a signaling pathway that includes PI3K and AKT. In view of this conservation, it seems justified to consider GSK3 as a potential target for the treatment of several CNS diseases [50]. Nevertheless, a word of caution is necessary concerning the use of inhibitors and their dosage in the light of the observed differential responses according to neuronal culture age and the differential effect over spine density. It is plausible that the failure of the GSK3-inhibition-based treatments for Alzheimer's disease may have been due to the ongoing hippocampal neurogenesis and, thus, cell stage heterogeneity [76].

## Supporting Information

S1 Fig GSK3, PI3K and AKT modify synapse number. Representative confocal images of larval motor neurons double immunostained against α-HRP (red) and nc82 antibody (green) from **A.** Control (*D42-Gal4*) **B.** GSK3 downregulation (*D42-Gal4/UAS-GSK3* ^*RNAi*^) **C.** Simultaneous overexpression of GSK3 and PI3K (*D42-Gal4/UAS-GSK3/UAS-PI3K*) **D.** Simultaneous downregulation of GSK3 and PI3K (*D42-Gal4/UAS-GSK3* ^*RNAi*^/*UAS-PI3K* ^*DN*^) **E.** Simultaneous overexpression of PI3K and downregulation of GSK3 (*D42-Gal4/UAS-PI3K/UAS-GSK3* ^*RNAi*^) **F.** Overexpression of AKT (*D42-Gal4/UAS-AKT*) and **G.** Simultaneous overexpression of AKT and downregulation of GSK3 (*D42-Gal4/UAS-AKT/UAS-GSK3* ^*RNAi*^). A high magnification view from the white square in **E** is shown in the upper right corner. Scale bars = 10 (**A-G**) and 2 μm (insert in **E**).
(TIF)

S2 Fig Dosage-dependent toxicity of GSK3 inhibitors. **A.** Representative confocal images of hippocampal neuronal cultures in control conditions and after treatment with the indicated concentrations of SB 415286 or AR-A014418. Neuronal density was quantified from high-resolution images comprising 40 individual pictures (8 x 5 fields, around 12 mm^2^). The pictures only show a detailed area of 6 mm^2^. Neuronal staining by MAP2B is shown in green and nuclear staining by DAPI is shown in red. DAPI marks all cells in the culture, astrocytes and neurons, while MAP2B marks only neurons. Scale bar = 50 μm. **B.** The effects of SB 415286 over neuronal survival were tested after 48 hours at three concentrations (10, 25 and 50 μM) and compared with control conditions (white histogram) in 12 DIV hippocampal neurons. **C.** Similar experiments were performed employing AR-A014418 (1, 10 and 50 μM) (n = 4 coverslips from 3 different cultures). Student’s two-tailed t-test, *p<0.05.
(TIF)
