# Supplementary figures and images for: GSK3β Inhibition Promotes Synaptogenesis in Drosophila and Mammalian Neurons
Source: PLoS One. 2015 Mar 12;10(3):e0118475. doi: 10.1371/journal.pone.0118475 (PMC4357437; doi:10.1371/journal.pone.0118475)

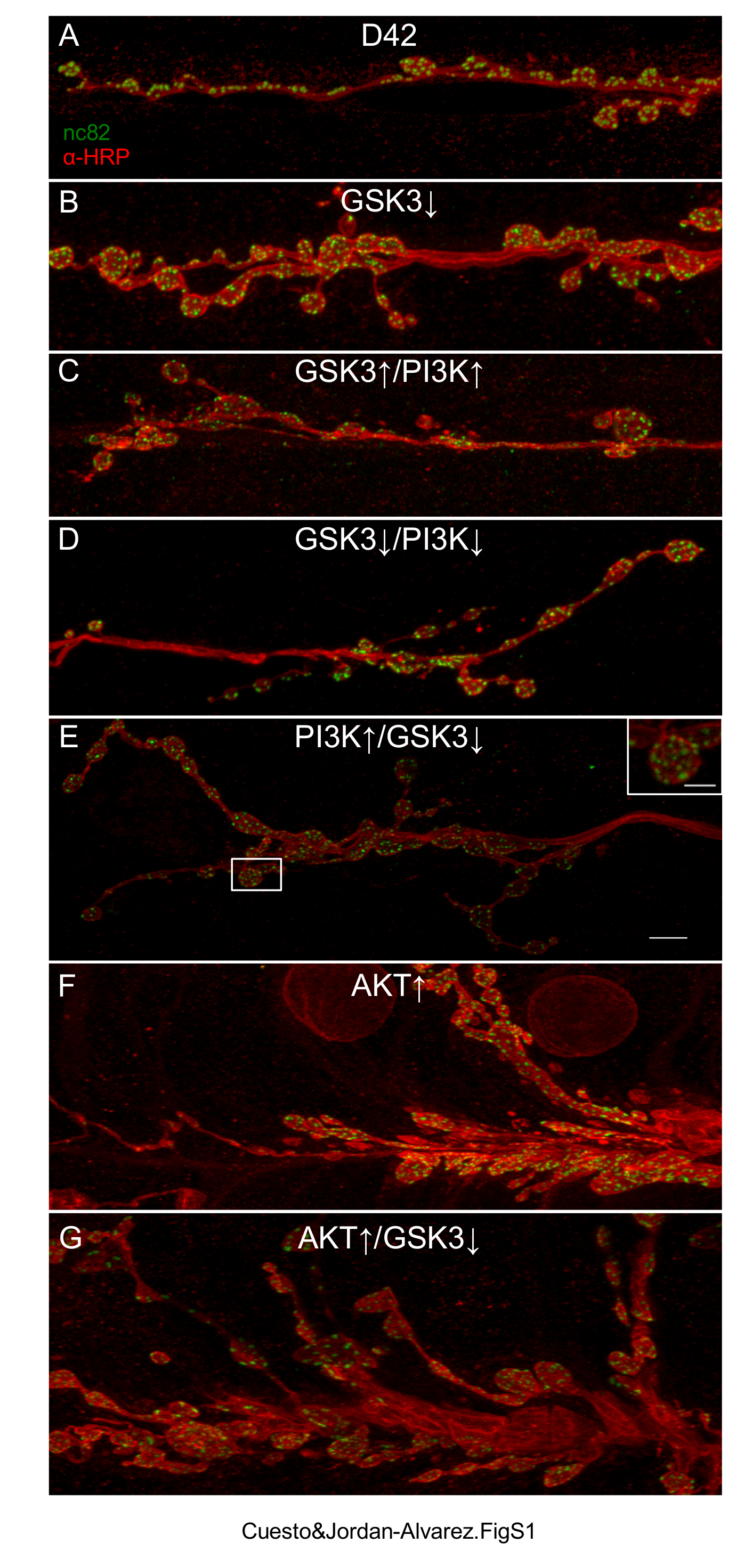

Supplement: S1 Fig — Representative confocal images of larval motor neurons double immunostained against α-HRP (red) and nc82 antibody (green) from A. Control (D42-Gal4) B. GSK3 downregulation (D42-Gal4/UAS-GSK3 RNAi) C. Simultaneous overexpression of GSK3 and PI3K (D42-Gal4/UAS-GSK3/UAS-PI3K) D. Simultaneous downregulation of GSK3 and PI3K (D42-Gal4/UAS-GSK3 RNAi/UAS-PI3K DN) E. Simultaneous overexpression of PI3K and downregulation of GSK3 (D42-Gal4/UAS-PI3K/UAS-GSK3 RNAi) F. Overexpression of AKT (D42-Gal4/UAS-AKT) and G. Simultaneous overexpression of AKT and downregulation of GSK3 (D42-Gal4/UAS-AKT/UAS-GSK3 RNAi). A high magnification view from the white square in E is shown in the upper right corner. Scale bars = 10 (A-G) and 2 μm (insert in E). (TIF) [file pone.0118475.s001.tif]

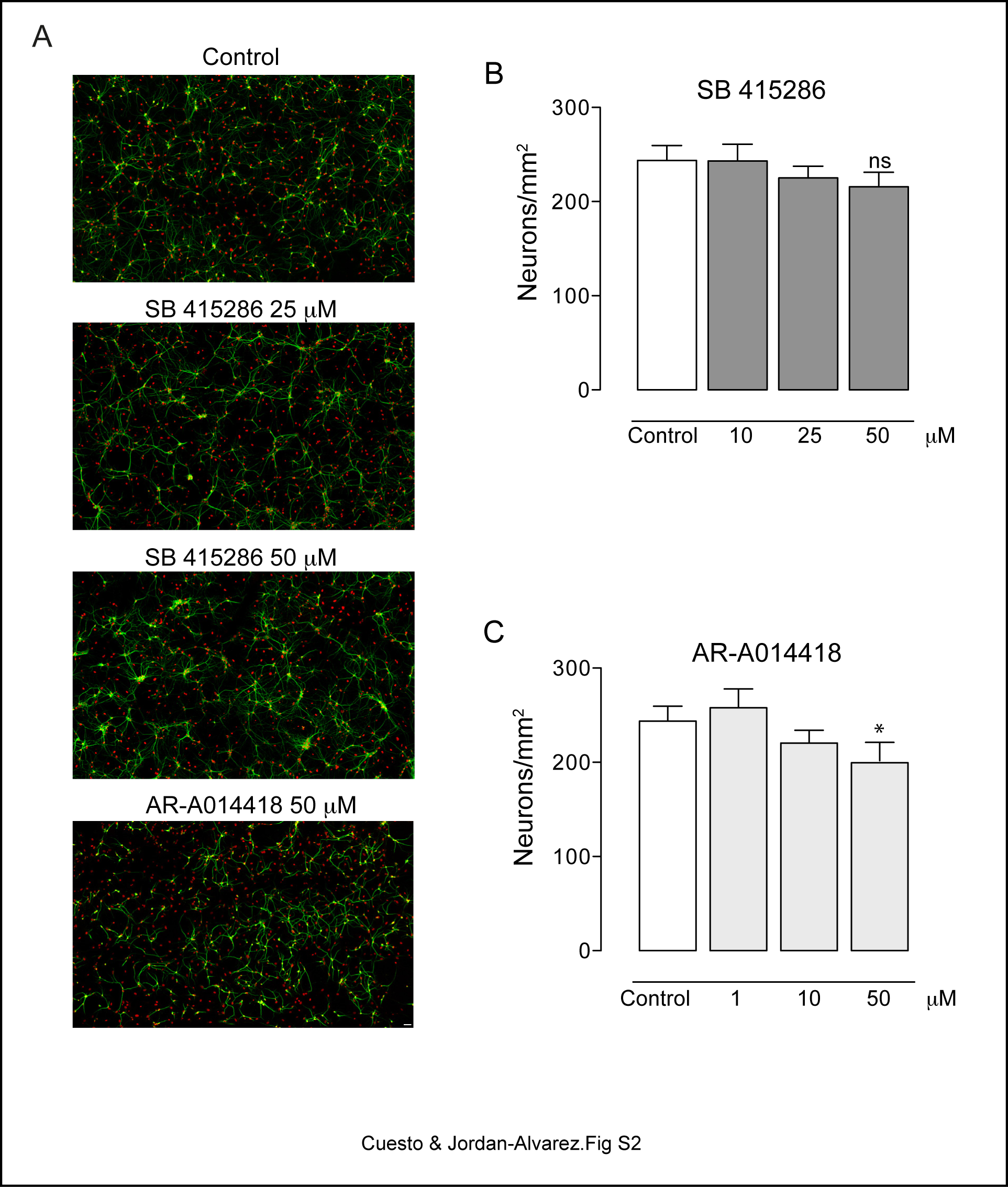

Supplement: S2 Fig — A. Representative confocal images of hippocampal neuronal cultures in control conditions and after treatment with the indicated concentrations of SB 415286 or AR-A014418. Neuronal density was quantified from high-resolution images comprising 40 individual pictures (8 x 5 fields, around 12 mm2). The pictures only show a detailed area of 6 mm2. Neuronal staining by MAP2B is shown in green and nuclear staining by DAPI is shown in red. DAPI marks all cells in the culture, astrocytes and neurons, while MAP2B marks only neurons. Scale bar = 50 μm. B. The effects of SB 415286 over neuronal survival were tested after 48 hours at three concentrations (10, 25 and 50 μM) and compared with control conditions (white histogram) in 12 DIV hippocampal neurons. C. Similar experiments were performed employing AR-A014418 (1, 10 and 50 μM) (n = 4 coverslips from 3 different cultures). Student’s two-tailed t-test, *p<0.05. (TIF) [file pone.0118475.s002.tif]
